# Securely sharing DSMB reports to speed decision making from multiple, concurrent, independent studies of similar treatments in COVID-19

**DOI:** 10.1017/cts.2022.387

**Published:** 2022-04-11

**Authors:** Natalie A. Dilts, Frank E. Harrell, Christopher J. Lindsell, Samuel Nwosu, Thomas G. Stewart, Matthew S. Shotwell, Jill M. Pulley, Terri L. Edwards, Emily Sheffer Serdoz, Katelyn Benhoff, Gordon R. Bernard

**Affiliations:** 1 Vanderbilt Institute for Clinical and Translational Research, Vanderbilt University Medical Center, Nashville, TN, USA; 2 Department of Biostatistics, Vanderbilt University Medical Center, Nashville, TN, USA; 3 Executive Committee for the Coordinated Approach for Emergency Studies

**Keywords:** Data and Safety Monitoring Boards (DSMBs), trial monitoring, data harmonization, safety monitoring, data sharing/pooling

## Abstract

**Introduction:**

As clinical trials were rapidly initiated in response to the COVID-19 pandemic, Data and Safety Monitoring Boards (DSMBs) faced unique challenges overseeing trials of therapies never tested in a disease not yet characterized. Traditionally, individual DSMBs do not interact or have the benefit of seeing data from other accruing trials for an aggregated analysis to meaningfully interpret safety signals of similar therapeutics. In response, we developed a compliant DSMB Coordination (DSMBc) framework to allow the DSMB from one study investigating the use of SARS-CoV-2 convalescent plasma to treat COVID-19 to review data from similar ongoing studies for the purpose of safety monitoring.

**Methods:**

The DSMBc process included engagement of DSMB chairs and board members, execution of contractual agreements, secure data acquisition, generation of harmonized reports utilizing statistical graphics, and secure report sharing with DSMB members. Detailed process maps, a secure portal for managing DSMB reports, and templates for data sharing and confidentiality agreements were developed.

**Results:**

Four trials participated. Data from one trial were successfully harmonized with that of an ongoing trial. Harmonized reports allowing for visualization and drill down into the data were presented to the ongoing trial’s DSMB. While DSMB deliberations are confidential, the Chair confirmed successful review of the harmonized report.

**Conclusion:**

It is feasible to coordinate DSMB reviews of multiple independent studies of a similar therapeutic in similar patient cohorts. The materials presented mitigate challenges to DSMBc and will help expand these initiatives so DSMBs may make more informed decisions with all available information.

## Introduction

Data and Safety Monitoring Boards (DSMBs) are charged with ensuring the safety of clinical trial participants and the validity and integrity of trial data [1]. The outbreak of the novel coronavirus SARS-CoV-2 pathogen, known as the COVID-19 pandemic, posed unique challenges to DSMBs overseeing studies of a disease not well characterized and treatments never tested in context [2]. In addition, by the summer of 2020 as the rate of COVID-19 related deaths in the United States surpassed 125,000 [3], the extended timeline to disseminate trial results to practitioners was a delay that investigators and the public could not afford. In the early months of the pandemic, as multiple clinical trials were initiated, individual DSMBs were deliberating in a vacuum of knowledge despite accruing information in seemingly identical trials. As an increasing array of data became available, DSMBs remained naïve to the information in larger, informative trials. In the absence of information, there is known potential for individual study data to produce an inaccurate signal for efficacy, futility, or harm, thereby increasing the risk of premature trial discontinuation [2, 4, 5]. Research indicates that the early termination rates for randomized control trials are between 10% and 12% [6, 7]. Reasons cited include insufficient accrual, findings of efficacy or toxicity, and safety concerns. A 2016 analysis of 249 discontinued randomized controlled trials revealed 18.5% were discontinued for early benefit or futility [8]. There is some evidence that stopping early for efficacy can substantially overestimate therapeutic benefit [9, 10].

DSMBs looking at a single study benefit from the context of other study findings in a similar therapeutic area to fully understand the scientific landscape. In fact, the Code of Federal Regulations Title 21 Section (c)(1)(i)(c)(ii) states, “The sponsor must report any findings from epidemiological studies, pooled analysis of multiple studies, or clinical studies… whether or not conducted by the sponsor, that suggest a significant risk in humans exposed to the drug” [11]. The FDA 2021 guidance for safety reporting notes the requirement for an aggregated analysis has the benefit to allow sponsors (and by extension DSMBs) to meaningfully interpret safety signals of investigational therapeutics [12]. Research suggests DSMBs should exercise patience when a small study shows positive or negative signal, especially if the signal from multiple studies is discordant [2, 4]. Conversely, DSMB confidence in reviewing stopping rules for a specific study can be enhanced by understanding there is directional concordance with other studies in the same therapeutic area [2, 4].

In our experience, DSMBs sometimes request prepublication trial results from similar studies so that decisions can be made based on the totality of available evidence. Consequently, the National Center for Advancing Translational Sciences (NCATS) Trial Innovation Network (TIN) investigators sought to formalize a method by which independent DSMBs, each overseeing independent studies of similar therapeutics in a similar disease setting, could share their data while maintaining autonomy, preserving their primary role in individual study oversight, and protect the confidentiality of the studies.

Extending beyond a mechanism for sharing DSMB reports, many clinical trials report a similar set of elements to the DSMB (e.g., accrual, adverse events, outcomes) creating an opportunity to combine data. Collating common reporting elements into a single, consistently formatted report has the potential to provide greater context and actionable information. We, therefore, sought to extend freely available visualization and data reporting tools to enable the generation of a harmonized DSMB report compiling information from *multiple* independent studies, with subsequent delivery to each study’s independent DSMB via a secure, digital platform **.** Combining a digital format with modern statistical graphics provides DSMB members with a tool to further evaluate individual-level data and to investigate the safety and efficacy signals from the participating studies without breaking any study’s blind.

Here, we describe the systems, processes, and tools that were developed to complete this DSMB Coordination (DSMBc) initiative. Challenges to the sharing of harmonized DSMB reports are described, as well as strategies for overcoming them.

## Methods

The DSMBc effort was initiated in July 2020 leveraging a set of Convalescent Plasma randomized-controlled trials (RCTs) as these studies were actively accruing and overseen by DSMBs that could benefit from the sharing of intermittent safety and efficacy data. From March 1, 2020 to June 1, 2020, thirty-three RCTs utilizing SARS-CoV-2 convalescent plasma for the treatment of Sars-CoV2 were identified on ClinicalTrials.gov as registered in the United States [13]. Initially, three RCTs were identified as being potentially informative to the overall drug effect question. The *Convalescent Plasma to Stem Coronavirus (CSSC-001)* (NCT04323800) outpatient trial enrolling asymptomatic high-risk subjects who experienced a close contact exposure to a person with COVID-19 in the past 120 hours, target *N* = 500 [14] and the *Convalescent Plasma to Limit Coronavirus Associated Complications (CSSC-004)* (NCT04373460) outpatient trial enrolling COVID-19 symptomatic subjects with a positive by RNA detection test for SARS-CoV-2, target *N* = 1344 [15] were both led by investigators at Johns Hopkins University. The third study, the *Passive Immunity Trial for Our Nation (PassItOnII)* (NCT04362176) enrolling symptomatic inpatient subjects with laboratory-confirmed SARS-CoV-2 infection, target *N* = 1000 [16] was led by investigators from Vanderbilt University Medical Center (VUMC). We initially selected the two CSSC trials for DSMBc as they were investigating the same therapeutic in comparable patient populations and were concurrently accruing. We posited that data from these two studies could be successfully harmonized as they were both outpatient studies with similar outcome measures.

In October 2020, a fourth international RCT was identified, the *CONvalescent Plasma for Hospitalized Adults With COVID-19 Respiratory Illness (CONCOR-1)* (NCT04348656) inpatient trial enrolling participants with confirmed SARS-CoV-2 infection receiving supplemental oxygen, target *N* = 1200 [17]. We added the CONCOR-1 trial as a direct comparator to the PassItOnII study since both were inpatient trials with similar accrual goals and outcome measures.

There was some overlap in personnel between the DSMBc initiative and the PassItOnII trial as disclosed in the Acknowledgements section of this manuscript. The CSSC studies shared a single DSMB but had independent data and safety monitoring plans. The PassItOnII and CONCOR-1 DSMBs were each fully independent.

### Initial Engagement

To obtain agreement from clinical trial principal investigators (PIs) and DSMB members, a DSMBc virtual meeting was scheduled with the PIs of the three initial trials (PassItOnII, CSSC-001 and CSSC-004). The goal of the DSMBc initiative was introduced, with emphasis that collaboration would be needed to share information, that transparency among studies would be paramount, the confidentiality of data would be critical, that allowing each study to achieve its own rigorous scientific answer was a priority, and sharing data as studies continued to accrue results (rather than only at the end of a given study) would be essential to DSMBc activities. A second virtual meeting was then held to introduce the project to the DSMB chairs of each trial. The result of these initial meetings was the development of a DSMBc process map to describe the three different phases needed for the DSMBc initiative: onboarding; secure data access; and generation/sharing of DSMBc harmonized reports. The process maps delineating responsibilities during each phase, the secure flow of data, and the process for sharing the DSMBc harmonized report are publicly available at https://rocket.app.vumc.org/index.php?doc_id=31371. The detailed approach for DSMBc data sharing procedures and the processes for report creation and verification are described in our Standard Operating Procedure (SOP), which are available in the supplementary materials [Supplemental Appendix 1].

### Regulatory and Contractual Agreements

The *DSMB Coordination for COVID-19* initiative was approved as exempt research under 45 CFR 46 104.4(d)(4) by the VUMC Human Research Protections Program [18]. Data Use Agreements (DUAs) were executed between VUMC and Johns Hopkins University to acquire copies of the CSSC-001/004 protocol, consent forms, case report forms (CRFs), data dictionaries, DSMB charters, and clinical trial data. Because the PassItOnII clinical trial was sponsored by VUMC, DUAs were not required for these study materials. Each study team reviewed the consent forms for the trials to confirm they allowed for the sharing of study data.

The DSMBc initiative did not overlap with any oversight activities that might be described in a DSMB Charter. The DSMBc serves as honest brokers, intended to support, and not supplant the usual DSMB operations. A DSMBc Report Sharing Agreement was developed and subsequently executed to address several key issues, such as how the DSMBc report would be distributed to the individual trial DSMBs, the data sharing and retention policy, the conflict-of-interest acknowledgement, the confidentiality policy, and a breach clause (a template is provided in Supplemental Appendix 2). The data retention statement indicated that the DSMBc could use the individual study data as long as necessary to implement, administer, and manage the harmonized reporting efforts of the DSMBc initiative, which could extend beyond the life of an individual study. The agreement also noted that the individual study data and reports would be retained up to 2 years following the conclusion of study recruitment and DSMB activities of all studies, at which point the study data would be destroyed unless otherwise directed by the FDA.

### Data Acquisition

To minimize the burden of providing trial data for the harmonized report, study teams were invited to upload their raw data to a central portal without modification for central harmonization. The time required to map data elements to a common data model had been identified as a barrier to participation during engagement activities, so DSMBc biostatisticians harmonized the data centrally.

The secure file upload application in the Research Electronic Data Capture (REDCap) system [19] was utilized by individual study personnel for secure data transfer. Managing REDCap user rights allowed us to restrict user access and permissions to ensure that blinded statisticians, including the one at VUMC, remained blinded to all data presented with treatment assignment. Acceptable formats for the uploaded data included R, SAS, SPSS, Stata, CSV, or Excel format. As appropriate, a file specifying the data format was also uploaded. In addition to the data, a copy of the study interim analysis report was requested. Alternative approaches for data sharing were considered, such as utilizing an established public (trial) data repository. This approach was not pursued because of confidentiality concerns and because it was not clear how to keep some team members blinded to treatment assignments while allowing others to be unblinded. While a public repository specifically suited to the needs of future DSMBc may become available, this was beyond the scope of this proof of concept.

Acquiring the protocol, case report forms, data dictionary, DSMB charter, and DSMB report shell well in advance greatly informed the process for harmonizing data, allowing the DSMBc biostatisticians to identify the relevant enrollment, eligibility, demographic, safety, and outcome variables for the final harmonized report. In addition, mock data from within the CSSC-004 trial electronic data capture system were provided to facilitate variable mapping. The mock data set allowed the statisticians to prepare the necessary code to harmonize in advance of receiving raw trial data, and we recommend the provision of the mock data file as an efficiency measure. Even given this advanced preparatory work, data sharing needed to occur at least three weeks prior to providing any DSMB with a harmonized report to ensure sufficient time to generate the harmonized report and review it with the statisticians of the collaborating trials.

### Statistical Analysis Approach

The goal for the DSMBc harmonized reports was not to replicate the individual study interim analysis plans, but to characterize those data that could be mapped across study groups, such as baseline data, safety data, and occasionally efficacy data. These were displayed using interactive statistical graphics offering both high-level summaries and individual patient data. The interactive, harmonized reports provided a synchronized review of data from multiple studies with the following items:

- Subject accrual, accounting for regions, countries, sites
- Descriptive statistics for baseline and longitudinal data
- Event report for binary events such as serious adverse events
- Number-at-risk report (declining denominators for longitudinal data)

The detailed harmonized reports featured interactive graphical elements to explore patterns as well as to read individual data off the graphics. Graphic elements include spike histograms, extended box plots, multiway dot plots, and trend line plots. The harmonized report was created with the hreport package in R (hbiostat.org/R/hreport), which can be used to create an interactive html report with R markdown [20].

To minimize the risk of inadvertently unblinding the data, the DSMBc Report Sharing Agreement explicitly stated that reports would be made available to the individual study DSMBs only during their closed meetings and only in executive session when attendance is limited to the voting DSMB members. To restrict distribution of the report outside of the individual DSMBs, the harmonized reports were presented in a format that prevented downloading and a statement was prominently displayed in the report that the data contained therein was unmonitored and interim and could not be downloaded or shared. Finally, the confidentiality statement in the DSMBc Report Sharing Agreement signed by all DSMB members stated that all data and materials submitted to the DSMBc group and DSMB members of participating studies will be kept confidential, will not be downloaded from the secure server on which they are provided, and will not be disclosed to anyone except as required by law, or to the extent necessary to evaluate the harmonized report. Each DSMBc and DSMB member of participating studies agreed to use appropriate safeguards to protect the confidentiality of shared data and prevent unauthorized use or access to the data.

To allow for secure access to the reports, the DSMBc honest brokers created a custom portal [21]. This allowed the DSMBc group to control user access to the reports through password protection and account management, ensure the successful file upload of reports, and allow for the sharing of user support materials (e.g., sample reports; How-To Quick Guides, and instructional videos). The DSMBc portal was designed to accommodate three different user roles with varying access rights and permissions (Table 1). The Administrator role had the broadest permissions and was limited to select members of the portal application development team. The Data Manager role was designated for DSMBc staff and allowed access to all projects and reports, but with more limited permissions. This was the role utilized by DSMBc biostatisticians who were responsible for generating and uploading harmonized reports. The Personnel role had the most restricted access and was limited to all other users, including the DSMB members, PIs, and study statisticians of the participating trials. Early feedback from DSMB members identified a need for a feature that would allow users to document comments and notes on their own individual digital copy of the harmonized report for later reference and archiving in the DSMBc file. In response, we developed a portal feature that supports both private and shared (among DSMB members) comments and notations.

**Table 1.** Data and Safety Monitoring Board Coordination (DSMBc) Portal user roles and permissions

	Administrator Member	Data Manager	Personnel
DSMBc portal permissions			
Access to all projects and reports	✓	✓	
Access to specific project and corresponding reports only			✓
Ability to manage users, projects, reports	✓		
Ability to add comments	✓	✓	✓
Ability to read all comments	✓	✓	
Ability to read public comments only			✓
Ability to update all comments	✓		
Ability to update own comments only		✓	✓
Ability to delete all comments	✓		
Ability to delete own comments only		✓	✓

### Integrating CONCOR-1

In October 2020, during the variable mapping of the two CSSC trials and the PassItOnII trial, the PI of the CONCOR-1 RCT expressed interest in participating in the DSMBc initiative. Within 9 weeks, the CONCOR-1 team formally agreed to participate, shared their study materials (protocol, data dictionary, consent forms, case report forms, and DSMB charter), confirmed preliminary DSMBc harmonized report data mapping, and shared a mock data set. However, it took an additional 11 weeks to execute a Data Use Agreement to allow for the sharing of actual trial data. During this time, the independent data and safety monitoring committee for CONCOR-1 recommended that the trial should stop enrollment as it had met the predefined threshold for futility. However, the CONCOR-1 PI was committed to sharing their final interim analysis data with the DSMBc initiative. The interim data were shared on 2 March 2021.

### Progress

**Fig. 1.**
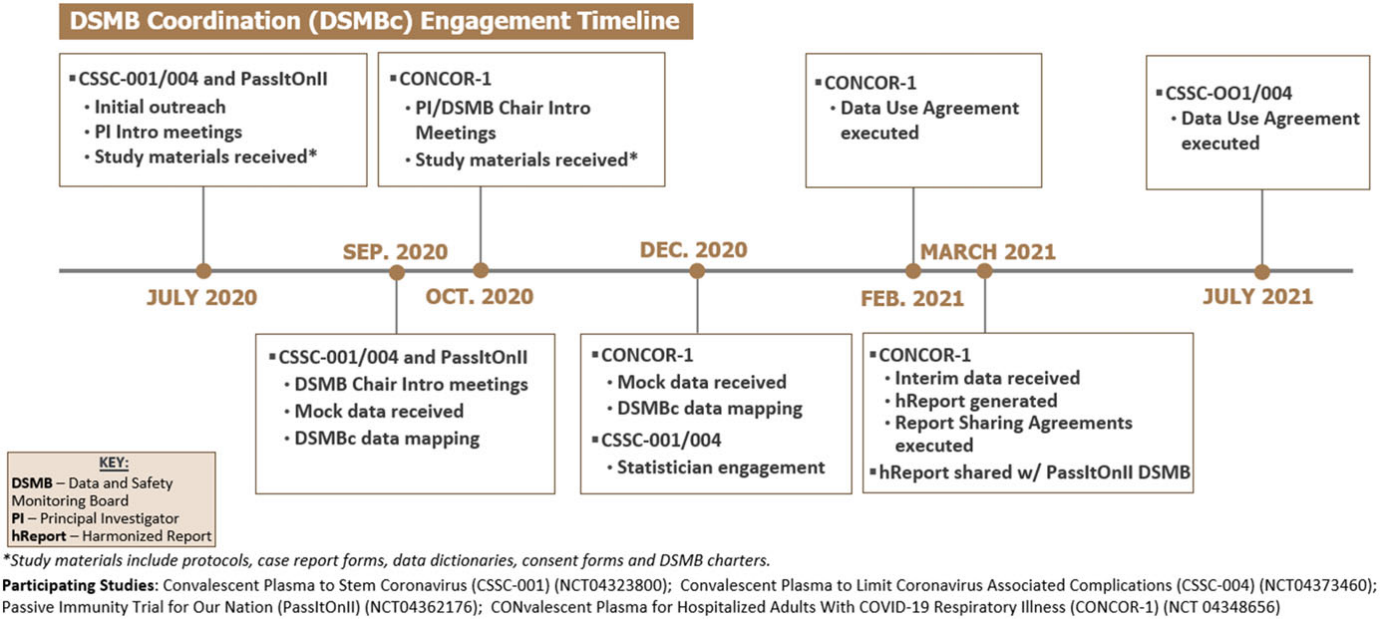
DSMBc engagement timeline.

**Fig. 2.**
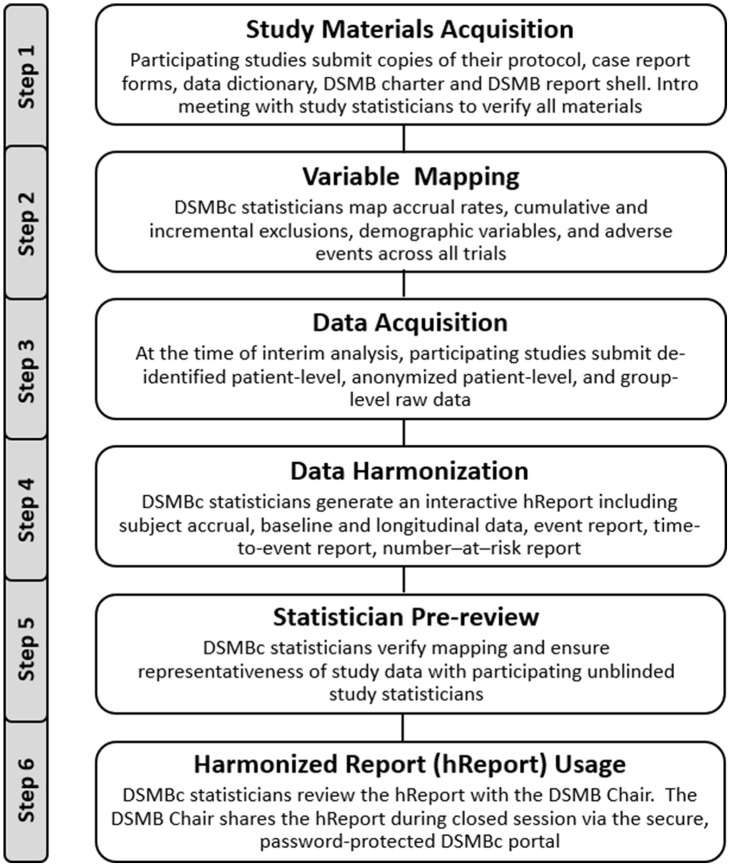
Data and Safety Monitoring Board Coordination (DSMBc) hReport process.

The DSMBc initiative successfully engaged the study teams and DSMBs of four randomized clinical trials investigating the use of convalescent plasma for the treatment of Sars-CoV2. Data Use Agreements were executed with three of the four trials; the fourth trial did not require a DUA as it was institutionally co-located. Protocols, consent forms, case report forms (CRFs), data dictionaries, and DSMB charters were acquired from all four trials. A timeline for the initiative is shown in Fig. 1, which shows our progress to date, and which demonstrates the complexity of coordinating DSMB review among multiple studies with discordant and unpredictable meeting schedules. A detailed visual timeline outlining key steps in the generation of a hReport is shown in Fig. 2.

At this time, all participating studies have closed to enrollment. The CONCOR-1 data were successfully harmonized with the PassItOnII data and the harmonized report shared with the PassItOnII DSMB in closed session prior to completing accrual. For the two CSSC studies, all of the administrative, regulatory, and contractual processes were completed and data mapping had been confirmed. However, due to the delayed execution of the JHU DUA, CSSC data were not harmonized since the proof of concept had already been demonstrated via the successful compilation and sharing of a harmonized report with a sitting DSMB-making decisions on a trial underway. It was determined the opportunity cost to dedicate more effort without the possibility of the harmonized report having any impact on an existing trial was too high. Due to meeting the threshold for futility, the CONCOR-1 DSMB completed their deliberations without reviewing a harmonized report, although they requested to review the report after the trial data were unblinded to provide feedback on its potential utility and whether it might have helped their decision making.

### DSMB Member Feedback

Of the three main DSMB reporting domains (baseline characteristics, safety, and outcomes), DSMB members reported that the additional safety data offered in the harmonized report was most helpful. DSMB members recommend careful attention be paid to whether the safety data being compared was being purposefully and actively tracked across each of the contributing studies; absent systematic collection of safety data, misinterpretation of safety signals would be likely. In addition, DSMB members commented that the baseline characteristics provided an opportunity to compare similarities and differences in the cohorts enrolled in the trials. Outcome data were reported to be the least helpful section.

The DSMBc initiative required the collaborative efforts of research teams, the implementation of administrative processes, and the development of new tools for data and report sharing and for generating harmonized reports. Our project demonstrates that all of these steps are feasible. Our experience also identified the considerable challenges that must be overcome for the benefits of DSMBc to be realized. We have summarized these challenges, and the stakeholders associated with emphasizing the challenges, in Table 2. The main solutions implemented to overcome these concerns were introductory meetings to detail the initiative, providing information and contractual agreements about data sharing and how data are managed and controlled, and the use of a DSMBc portal serving as a secure honest broker.

**Table 2.** Stakeholder-identified barriers and solutions to data sharing for harmonized reports

	DSMB Chair	DSMB Member	Principal Investigator	Study Statistician
Concerns				
Security of data	✓			
Confidentiality	✓	✓		
Unblinding	✓		✓	✓
Public data sharing			✓	✓
Authorship			✓	✓

Key: DSMB – data and safety monitoring board.

## Discussion

Coordinating DSMB review among independent DSMBs of independent studies with a harmonized DSMB report containing information from all studies is feasible. This work provides a starting place for discussions about the additional value DSMBc might provide given the additional effort that it requires.

### Data to Include

Our DSMBc initiative was a coordinated effort between research institutions, each of which was leading a trial on the effect of convalescent plasma in COVID-19 patients. Implicitly, a prerequisite for a DSMBc initiative is that at least two trials simultaneously investigating the same (or similar) intervention in a comparable patient population. If such a setting exists, as it did during the COVID-19 pandemic, then DSMBc provides a mechanism for DSMB members to examine summaries about the patient characteristics, safety events, and outcomes from the ongoing or recently completed comparable trials. Potentially, DSMBc can provide beneficial aggregated safety data for the review of therapeutics often tested in multisite trials, including PD-1 inhibitors, blood pressure lowering therapies, and oxygen targets in mechanically ventilated patients. In addition, the sharing of data between US only trials and non-US studies can facilitate harmonized reviews of baseline characteristics and safety events to inform decision making in real time. However, we acknowledge the DSMBc approach is unlikely to fit all programs of research. For example, whether the benefits of a DSMBc would be realized in an industry-sponsored program of drug development is unclear; the proprietary and coordinated nature of such research programs may not warrant a DSMBc approach. Conversely, the process might be especially well suited to broad programs of alike research supported by agencies such as the NIH. While institutional DSMBs often oversee multiple trials, we are not aware of efforts toward data harmonization and joint reporting where appropriate.

At the outset of the current DSMBc initiative, our contention was that providing the additional data would facilitate cross-study comparisons and help inform DSMB decision making about safety and efficacy in individual trials. Our results stem from a single DSMBc initiative and thus reflect only an exemplar case study, but initial feedback indicated that the safety reporting was of most value to individual DSMBs. This might be expected given that DSMBs are generally charged to prioritize safety above efficacy [22, 23]. Variables related to safety events, while not as straightforward as baseline characteristics, are reasonably standardized. It is generally the case that adverse events are classified by event type, severity, body system, and organ classes using one of a number of standardized classification schema (MedDRA, CTCAEs). Assuming participating studies are continually cleaning and coding their adverse events, then the safety data can be readily harmonized between studies using available mappings between these existing classification schemes.

While our proof-of-concept provided harmonized reporting for baseline characteristics, safety events, and outcomes, it is notable that many of the administrative and collaborative challenges stemmed from sharing and reporting only the outcome data. These barriers can be minimized with the implementation of multisite trials and/or the establishment of a minimum set of outcome measures that are interoperable upfront. Notwithstanding, if our initial feedback reflects majority opinion about the relative value of outcome data, then future DSMBc initiatives may be simplified by excluding outcome data from the harmonized reports altogether. This would also simplify the mapping processes because baseline characteristics tend to be organized in a straightforward, easily harmonized structure amenable to simple summarization. This could reduce the burden on the DSMBc honest brokers and the trial statistical teams as they attempt to reconcile the data sets. However, excluding outcome data from harmonized reports prevents an assessment of the risk to benefit ratio among the participating trials.

There are multiple aspects to sharing and summarizing outcome data that make it more challenging than the other types of data. First, endpoints are often different between studies. While observing directional trends in a range of outcomes across studies is informative, summarizing overall observed effect in a meaningful way is challenging without being able to pool outcome data for analysis. Second, the definition of study endpoints can involve nuances in data collection and derivation, especially as it relates to missing data or censoring. This requires extra time and effort from the study statisticians and the DSMBc honest brokers as they work together to verify the endpoints are calculated correctly. We also note that outcome data are more challenging to share, in part because independent DSMBs become privy to unblinded data used to support a trial’s decision-making process, creating a sense of unease. This is exacerbated because study PIs and statisticians note that that such data should be reported as accurately as possible and interpreted with caution, and there is concern an external DSMB does not have that context. This added sense of stewardship for the outcomes may create an obstacle for participating in future DSMBc initiatives. Since outcome data are putatively less valuable to participating DSMB members, we recommend DSMC initiatives focus first on safety and baseline characteristics.

### People

Successful implementation of the DSMBc initiative required work to foster connections with study teams and individual study DSMB leaders. We found it crucial to obtain support not only from the study team and DSMB members but also from the trial statisticians charged with stewardship over the data. Indeed, in failing to do this, we were substantially delayed and ultimately unable to compile data from two studies into a harmonized report. We suggest that engaging all participating statisticians at the beginning of the harmonization process will help to facilitate data stewardship discussions as well as improve the honest broker’s understanding of the data elements.

### Processes

We have provided our standard operating procedure so others may adapt our processes to their own purposes (Supplemental Appendix 1). We believe setting expectations for a DSMB at the outset of a study, such as by describing DSMBc activities in the DSMB charter, may help to facilitate uptake of data harmonization efforts. A major concern for the DSMBc initiative was ensuring consent for data to be used for the proposed purpose, and that confidentiality could be maintained. We recommend that clinical trials consider including language in the consent form that allow for efforts to harmonize trial data and oversight. This will mitigate the need for consent form revisions, submission of amendments to Institutional Review Boards, or re-consenting of subjects to allow for such data sharing. In addition, we also recommend that trials adopt common data elements where they exist (i.e., common variable labels, levels, names and definitions). The use of data standards as proposed by the Clinical Data Interchange Standards Consortium (CDISC) [24, 25] could facilitate harmonization activities and decrease the effort required to map variables across disparate trials, as well as limiting the introduction of errors due to the assumptions made in the harmonization process. Of course, if comparable trials adopt the same electronic data capture system with the same case report form, further efficiencies can be achieved in the harmonization.

Our experience with DSMBc has resulted in a set of proposed processes recommendations for future work in this domain. A list of these recommendations and the benefits they provide in the data harmonization process can be found in Table 3.

**Table 3.** Recommendations for future DSMB Coordination (DSMBc) efforts

Participating trial recommendations	Harmonization benefits
Include DSMBc activities in the DSMB charters	- Establishes DSMB expectations upfront - Facilitates uptake of the initiative
Adopt the same case report forms	- Increases efficiency in the harmonization of relevant enrollment, eligibility, and demographic data
Adopt the same electronic data capture system	- Increases efficiency in the harmonization of relevant safety data and outcome variables
Include data sharing language in the consent forms	- Ensures participant consent for use of trial data for DSMB Coordination and that confidentiality will be maintained
Establish a minimum set of interoperable outcome measures	- Ensures the interpretation of outcome data is preserved as data are collected and shared across different technical platforms. - Reduces the effort needed for data cleaning and preprocessing
Early and ongoing engagement with all participating statisticians	- Facilitates data stewardship discussions as well as improves the honest broker’s understanding of the data elements - Facilitates the verification that endpoints are calculated correctly
Adopt common data elements (variable labels, levels, names definitions)	- Facilitates harmonization activities and decreases the effort required to map variables across disparate trials. - Limits the introduction of errors due to the assumptions made in the harmonization process
Utilize standardized classification schema (e.g., Medical Dictionary for Regulatory Activities (MedDRA); Common Terminology Criteria for Adverse Events (CTCAE))	- Facilitates harmonization of safety data and decreases the effort required to map variables across disparate trials - Limits the introduction of errors due to the assumptions made in the harmonization process
Exclude Outcome data; focus on safety and baseline data	- Reduces extra time and effort to verify the endpoints are calculated correctly - Eliminates the challenge of summarizing overall observed effect in a meaningful way without being able to pool outcome data for analysis - Minimizes DSMB member unease with the sharing of unblinded data used to support a trial’s decision-making process - Eliminates inaccurate interpretation of outcome data by external DSMB members who do not have context - Safety data identified as most beneficial by DSMB members

Key: DSMB – Data and Safety Monitoring Board.

### Products

We approached the DSMBc initiative as a potential improvement to clinical trial efficiencies outside of the pandemic context as much as within it. Therefore, we developed processes using familiar and available infrastructure as much as possible. REDCap was used as a file repository, securely housing data use agreements and study data sets. Interactive harmonized reports utilizing statistical graphics were developed to provide synchronized reviews of data from multiple studies. An example of a DSMBc hReport is available in the supplementary materials [Supplemental Fig. 1]. The initiative did require the development of the secure DSMBc portal for report management and sharing, in addition to the expansion of the hreport package, both of which are now available to others seeking to improve trial efficiencies with a secure report management tool.

## Conclusion

It is possible to share patient-level data between independent DSMBs of ongoing, independent clinical trials. The process was developed using an iterative process and is presented here as a scalable framework for future studies. The next step is to explore opportunities for leveraging DSMBc outside of the pandemic context to potentially shorten the evidence to practice timeline.

## Supplementary material

For supplementary material accompanying this paper visit https://doi.org/10.1017/cts.2022.387. click here to view supplementary material
