# Supplementary material for: Securely sharing DSMB reports to speed decision making from multiple, concurrent, independent studies of similar treatments in COVID-19
Source: J Clin Transl Sci. 2022 Apr 11;6(1):e49. doi: 10.1017/cts.2022.387 (PMC9120618; doi:10.1017/cts.2022.387)

**SUPPLEMENTAL MATERIALS**

**Contents**

1. SUPPLEMENTAL APPENDICES
   1. Supplemental Appendix 1. DSMB Coordination (DSMBc) Standard Operating Procedures
   2. Supplemental Appendix 2. DSMB Coordination (DSMBc) Report Sharing Agreement
2. SUPPLEMENTAL FIGURES
   1. Supplemental Figure 1. Example of Selected Sections of a Harmonized Report (hReport)
3. **SUPPLEMENTAL APPENDICES**

Supplemental Appendix I

DSMB Coordination (DSMBc) Standard Operating Procedures

To improve participant safety and maximize scientific benefit, the Data Safety Monitoring Board Coordination (DSMBc) initiative will create visualization and data reporting tools that will enable synchronized review of *multiple* studies by a single DSMB entity**.** The organizing principle is that there are many analytical and reporting methods that many clinical trials studying the same intervention have in common (e.g., patient accrual reporting). Having high-level functions implementing these procedures in a common way saves a great deal of time. In addition, by using modern statistical graphics, DSMBs can be provided more digestible information to assist in their reviews as they safeguard the interests of study participants, assess the safety and efficacy of study procedures, and monitor the overall conduct of the study.

**hReport Definition**

Harmonized Reports (hReport): Visualization and data reporting tools to allow for quick comparisons of baseline distributions, safety signals, and efficacy outcomes across multiple clinical trials investigating a similar therapeutic.

**hReport Creation and Access**

For any study identified as being potentially informative to the overall intervention effect question, case report forms will be shared with the DSMB Coordination (DSMBc) biostatisticians. The DSMBc biostatisticians will map key risk and outcome variables between the case report forms so that when it comes time for any trial DSMB to meet, the request for information from across studies can be simplified and expedited. At the time of data lock for a study, the study will be asked to submit key risk and outcome variables for the relevant treatment groups to the DSMBc (under appropriate regulatory agreements and using appropriate technical systems to ensure security of this information). Upon receipt of clinical trial raw data, the DSMBc statisticians will create a single harmonized report (hReport), which has been developed using the R language and provides high level tools to support DSMB reporting.

The interactive hReport will include, but may not be limited, to the following:

- Subject accrual, accounting for regions, countries, sites
- Descriptive stats for baseline and longitudinal data
- Event report
- Time-to-event report
- Number-at-risk report (declining denominators for longitudinal data)

These hReports will be made available to the individual study DSMBs during their closed meetings, and only in executive session when attendance is limited to DSMB members only. DSMBc statisticians will be available prior to individual study DSMBc meetings to review hReports with the DSMB Chair and answer any questions about the presented data. Updated hReports will be generated multiple times over the life of the study.

Safety information, efficacy estimates, and hReports are not to be shared outside of the individual DSMBs, as hReports contain limited amounts of embedded raw patient data in addition to summary data. **hReports are not to be downloaded or shared**, and all other expectations set forth by the confidentiality statement in the Principles of Operation shall be followed.

hReports are only accessible via the custom portal created by Vanderbilt University Medical Center and are password protected. Additional information and user support materials (sample hReports, How-To guides, instructional videos) can also be found within this portal.

**Ongoing Communication**

The DSMBc’s work (including hReports) is an iterative process, and the team welcomes feedback from the individual study DSMB Chairs to improve operations to ensure hReports can best inform the decisions of the individual study DSMBs. The DSMBc Team will complete debriefs with DSMB Chairs to discuss possible process improvements.

Supplemental Appendix II

DSMB Coordination (DSMBc) Report Sharing Agreement

Mission of the DSMBc initiative

The mission of the DSMBc initiative is to harmonize data across trials of similar interventions to provide information to assist individual study DSMBs in their reviews as they safeguard the interests of study participants, assess the safety and efficacy of study procedures, and monitor the overall conduct of the study.

**INTRODUCTION**

To improve participant safety and maximize scientific benefit, the goal of this effort is to pilot a novel Data Safety Monitoring Board (DSMB) support plan, creating visualization and data reporting tools that will enable synchronized review of *multiple* studies by a single DSMB entity**.** The organizing principle is that there are many analytical and reporting methods that many clinical trials have in common (e.g., patient accrual reporting). Having high-level functions implementing these procedures in a common way saves a great deal of time. In addition, by using modern statistical graphics, DSMBs can be provided more digestible information.

For any study identified as being potentially informative to the overall intervention effect question, case report forms will be shared with the DSMB Coordination (DSMBc) biostatisticians. The DSMBc biostatisticians will map key risk and outcome variables between the case report forms so that when it comes time for any trial DSMB to meet, the request for information from across studies can be simplified and expedited. At the time of data lock for a study, the study will be asked to submit key risk and outcome variables for the relevant treatment groups to the DSMBc (under appropriate regulatory agreements and using appropriate technical systems to ensure security of this information).

Individual trial data will be combined into a single harmonized report (hReport). An individual trial DSMB will be able to use the hReport to investigate safety and efficacy signals from participating studies in order to inform their decisions at the time of review.

DEFINITIONS

**Data and Safety Monitoring Board (DSMB)**: An advisory group of experts/investigators with a mandate to periodically review and evaluate safety data during a clinical trial.

**Harmonized Reports (hReport)**: Visualization and data reporting tools to allow for quick comparisons of baseline distributions, safety signals, and efficacy outcomes across multiple clinical trials investigating a similar therapeutic.

**Principal Investigator**: The investigator who takes overall responsibility for, and is accountable for, all activities of the study.

**Study Biostatistician:** The biostatistician who is responsible for providing interim reports to the DSMB.

PROCEDURES

**Confidentiality**

All data and materials submitted to the DSMBc group and DSMB members of participating studies will be kept confidential and will not be disclosed to anyone except as required by law, or to the extent necessary to evaluate the hReport. The DSMBc will maintain all information in a secure environment and DSMB members will not download or create copies of data or reports.

Each DSMBc and DSMB member of participating studies shall utilize appropriate safeguards to protect the confidentiality of shared data and prevent unauthorized use or access to the data. Each DSMB member agrees to promptly notify the DSMBc group if they become aware of any breaches of confidentiality and to cooperate fully in any efforts to remediate the breach. If so instructed, DSMB members shall return data files or destroy the data files.

DSMBc Data Sharing

The individual study teams will provide copies of their protocol, a blank consent form, case report forms, data dictionary, and DSMB charter. The study’s DSMB report shell should also be provided. This should be done well in advance of attempts to harmonize data, as these documents will greatly inform the process for harmonizing data.

When data are ready to be shared, the study biostatisticians or data management teams will upload clinical trial analysis files using a secure system provided by the DSMBc, typically REDCap. The data can be provided in R, SAS, SPSS, Stata, CSV, or Excel format. As appropriate, a file specifying the data format should be uploaded. This should occur at least one week prior to expecting these data to be included in any hReport.

DSMBc Harmonized Report

Upon receipt of clinical trial raw data, the DSMBc statisticians will generate an hReport that that will enable synchronized review of multiple studies by each individual study DSMB. These hReports will be made available to the individual study DSMBs during their closed meetings, and only in executive session when attendance is limited to DSMB members only.

The interactive hReport will include, but may not be limited, to the following:

• subject accrual, accounting for regions, countries, sites

- descriptive stats for baseline and longitudinal data

• event report

• time-to-event report

• number–at–risk report (declining denominators for longitudinal data)

DSMBc Data Retention

The DSMBc will use the individual study data to implement, administer and manage the harmonized reporting efforts of the DSMBc initiative. The individual study data and hReports will be retained for 2 years following the conclusion of individual study recruitment and final DSMB activity, unless a longer period is requested by the FDA.

RESPONSIBILITIES

DSMB responsibilities

The individual study DSMB responsibilities are outlined in each study DSMB charter. For the purposes of DSMBc, the individual study DSMBs take on no additional responsibility except to maintain confidentiality of the additional information. hReports will be provided in the hopes of supplementing the DSMB understanding of patient safety, validity, and scientific merit of the study.  The hReports are provided as is, and the DSMBc does not warrant the accuracy of data provided to the DSMBc for the creation of the hReports.

DSMBc responsibilities

The DSMBc will provide hReports to the individual study DSMBs. The DSMBc will not monitor the safety of current and future participants or ensure that participants are not exposed to undue risk. The DSMBc will not provide recommendations regarding the continuing safety, validity, and scientific merit of the study.

Conflict of Interest

Prior to the sharing of DSMBc hReports, the individual study DSMB members for each study will disclose any potential conflicts of interest and complete a Conflict of Interest Statement. This shall include potential conflicts with all studies included in the hReport. *(If any of the following questions are answered with a 'yes' please attach all relevant material to this form.)*

| □ Yes | □ No | I, or a family member, is key personnel on a study participating in DSMBc |
| --- | --- | --- |
| □ Yes | □ No | I, or a family member, have previously received any payments such as salary, consulting fees, gifts etc. from the Principal Investigator, sub-Investigators or other persons associated with the conduct of any participating trials. |
| □ Yes | □ No | I, or a family member, has equity in the form of stock, stock options, real estate, loans, or another investment or ownership interest in any research involved with participating trials. |
| □ Yes | □ No | I, or a family member, hold a management position such as board member, director, officer, partner or trustee with a company related to this research. |
| □ Yes | □ No | I, or a family member, own or have intellectual property rights on a patent, patent application, or copyright whose value may be affected by the outcome of research conducted. |
| □ Yes | □ No | I, or a family member, is involved with the design, execution or analysis of any clinical trials conducted by a company related to this research. |

I acknowledge that I am responsible for notifying the Principal Investigator promptly if:

any changes occur in the information cited above during my role as a member of the Data Safety Monitoring Board for this study.

OR

I discover that an organization with which I have a relationship meets the criteria for a conflict of interest.

**DSMB Member Agreement**

As a member of the Data Safety Monitoring Board (DSMB) for a participating study, I agree to adhere to the terms and procedures described in this Principles of Operations.

|  |  |  |
| --- | --- | --- |
| *Signature* |  | *Date* |
|  |  |  |
|  |  |  |
| *Printed Name* |  |  |

1. **SUPPLEMENTAL FIGURES**

**Supplemental Figure 1.**
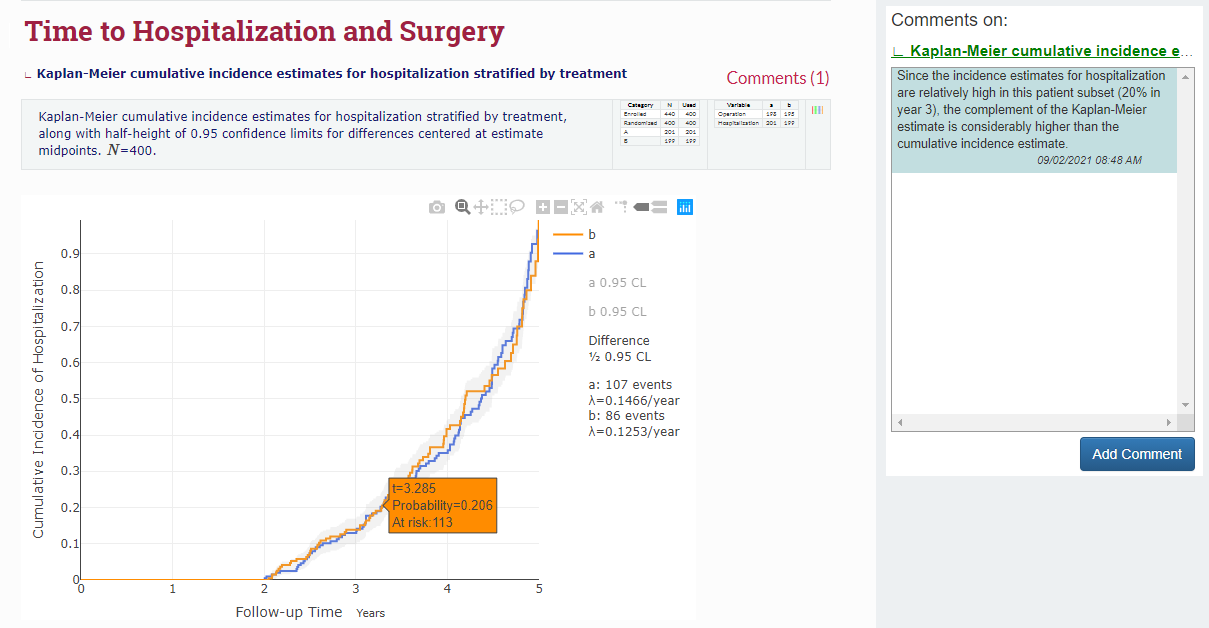
Example of Selected Sections of a Harmonized Report (hReport)


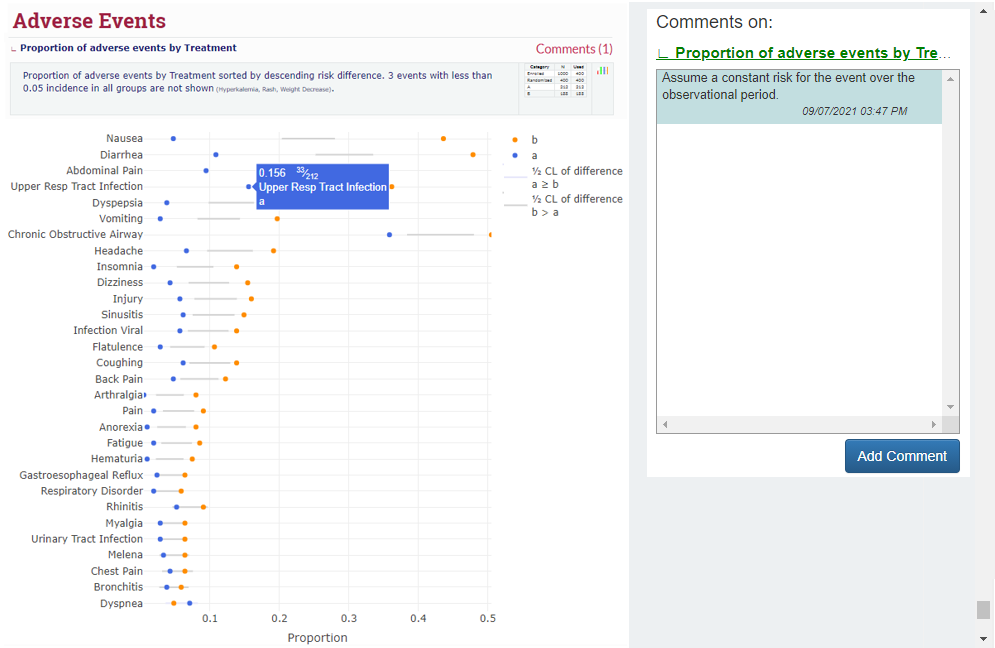

Supplement: Supplementary file 1 [file S2059866122003879sup001.docx]
